# GVHD-derived plasma as a priming strategy of mesenchymal stem cells

**DOI:** 10.1186/s13287-020-01659-x

**Published:** 2020-04-16

**Authors:** Amandda Évelin Silva-Carvalho, Leane Perim Rodrigues, Josiane Lilian Schiavinato, Marcos Rodrigo Alborghetti, Gustavo Bettarello, Belinda Pinto Simões, Francisco de Assis Rocha Neves, Rodrigo Alexandre Panepucci, Juliana Lott de Carvalho, Felipe Saldanha-Araujo

**Affiliations:** 1 Laboratório de Farmacologia Molecular, Departamento de Ciências da Saúde, Universidade de Brasília, Brasilia, DF Brazil; 2 Laboratório de Hematologia e Células-Tronco, Departamento de Ciências da Saúde, Universidade de Brasília, Brasilia, DF Brazil; 3 Programa de Pós-graduação em Ciências Genômicas e Biotecnologia, Universidade Católica de Brasília, Brasilia, DF Brazil; 4 Laboratório de Hematologia, Hospital das Clínicas da Faculdade de Medicina de Ribeirão Preto, Universidade de São Paulo, Ribeirao Preto, SP Brazil; 5 Laboratório de Bioquímica e Química de Proteínas, Departamento de Biologia Celular, Universidade de Brasília, Brasilia, DF Brazil; 6 Unidade de Transplante de Medula Óssea, Instituto de Cardiologia do Distrito Federal, Brasilia, DF Brazil; 7 Laboratório de Biologia Funcional (LFBio), Centro de Terapia Celular (CTC), Hemocentro de Ribeirão Preto, Rua Tenente Catão Roxo, Ribeirão Preto, SP 2501 Brazil; 8 Programa de Pós-graduação em Ciências Médicas, Faculdade de Medicina, Universidade de Brasília, Brasilia, DF Brazil

**Keywords:** Mesenchymal stem cells, Lymphocytes, Graft-versus-host disease, Immunomodulation, Mesenchymal stem cell priming

## Abstract

**Background:**

Mesenchymal stem cell (MSC) therapy is an important alternative for GVHD treatment, but a third of patients fail to respond to such therapy. Therefore, strategies to enhance the immunosuppressive potential of MSCs constitute an active area of investigation. Here, we proposed an innovative priming strategy based on the plasma obtained from GVHD patients and tested whether this approach could enhance the immunosuppressive capacity of MSCs.

**Methods:**

We obtained the plasma from healthy as well as acute (aGVHD) and chronic (cGVHD) GVHD donors. Plasma samples were characterized according to the TNF-α, IFN-γ, IL-10, IL-1β, IL-12p40, and IL-15 cytokine levels. The MSCs primed with such plasmas were investigated according to surface markers, morphology, proliferation, mRNA expression, and the capacity to control T cell proliferation and Treg generation.

**Results:**

Interestingly, 57% of aGVHD and 33% of cGVHD plasmas significantly enhanced the immunosuppressive potential of MSCs. The most suppressive MSCs presented altered morphology, and those primed with cGHVD displayed a pronounced overexpression of ICAM-1 on their surface. Furthermore, we observed that the ratio of IFN-γ to IL-10 cytokine levels in the plasma used for MSC priming was significantly correlated with higher suppressive potential and Treg generation induction by primed MSCs, regardless of the clinical status of the donor.

**Conclusions:**

This work constitutes an important proof of concept which demonstrates that it is possible to prime MSCs with biological material and also that the cytokine levels in the plasma may affect the MSC immunosuppressive potential, serving as the basis for the development of new therapeutic approaches for the treatment of immune diseases.

## Background

Mesenchymal stem cells (MSCs) are adult multipotent stem cells that display several properties potentially applicable to the cell therapy field, including their antibacterial, antiapoptotic, proangiogenic, regenerative, and immunomodulatory capacity [1]. It is well known that MSCs can interact and modulate the function of virtually all cells of the immune system, primarily through contact-dependent mechanisms involving the participation of the vascular cell adhesion protein 1 (VCAM-1), intercellular adhesion molecule 1 (ICAM-1), and programmed death-ligand 1 (PDL-1) [2, 3]. A broad range of biologically active molecules that are secreted by MSCs also integrates the immunomodulatory arsenal of such cells. In this context, the major immunosuppressive molecules produced by MSCs are HGF, TGF-β [4], IDO [5], PGE2 [6], IL-6 [7], IL-10 [8], semaphorin-3A, galectin (Gal)-1 [9], Gal-9 [10], and adenosine [11].

As knowledge regarding the immunosuppressive potential of MSCs accumulates, some obstacles still remain to be overcome, such as the high number of MSCs required for clinical application and optimizing their therapeutic effect to ensure a more homogenous clinical outcome. Considering MSC application for the treatment of graft-versus-host disease (GvHD), several clinical trials have already been executed and consistently point towards the safety and efficacy of MSC therapy for GVHD [12–19]. Nevertheless, about a third of patients fail to respond to treatment, especially adults.

Aiming to tackle such therapeutic limitation, several researchers have investigated priming (a.k.a licensing) strategies in order to enhance the immunosuppressive potential of MSCs, including peptides [20], agonists for Toll-like receptors [21], and proinflammatory recombinant factors [22], among which IFN-γ is the most intensively investigated molecule so far [23–25]. Still, in the first clinical investigation of IFN-γ-primed MSCs, data was disappointing, justifying further studies [26].

Here, we tested an innovative priming strategy by submitting the MSCs to the plasma from GVHD patients and investigated whether the inflammatory milieu generated by GVHD influences the molecular mechanisms of MSCs to suppress the immune response and how different characteristics of the plasma and the primed MSCs correlate to the immunosuppressive capacity of such cells.

## Methods

### Plasma collection and preparation

Peripheral blood was collected from patients previously treated with bone marrow transplantation that developed acute GVHD (aGVHD, *n* = 7) and chronic GVHD (cGVHD, *n* = 12) at the Institute of Cardiology of Federal District and at the University Hospital of the Medical School of Ribeirão Preto (University of São Paulo, Brazil). Samples were identified as GA 1-7 and GC 1-12, respectively. In addition, the plasma from 8 healthy donors was obtained and pooled to serve as the control samples. In order to obtain a biologic material composed of soluble factors only without platelet interference, we depleted the platelet content of plasmas. For this, samples were first centrifuged at 400*g* for 10 min, and the upper portion of the plasma with platelets was removed. Then, the samples were centrifuged again at 800*g* for 10 min, and the plasma was collected [27].

### Enzyme-linked immunosorbent assay

In order to better characterize the inflammatory profile of the GVHD samples, we quantified the following factors: TNF-α, IFN-γ, IL-10, IL-1β, IL-12p40, and IL-15 by ELISA, following the manufacturer’s instructions (ImmunoTools). The absorbance of each well was measured at 450 nm using the automatic microplate reader DTX 800 Multimode Detector (Beckman Coulter). Samples were diluted 1:2 with blocking buffer and analyzed in duplicates.

### MSC culture and characterization

The study protocols were approved by the Institutional Ethics Committee (protocol 64079216.3.0000.0030), and written informed consent was obtained from all participants. MSCs were obtained from three healthy donors following a lipoaspiration procedure. The cells were cultured in alpha-Minimum Essential Medium (α-MEM) supplemented with 15% fetal bovine serum (FBS—HyClone, Logan, UT, USA), 2 mM glutamine, and 100 U/mL penicillin/streptomycin (Sigma, St. Louis, MO, USA), at 37 °C and 5% CO_2_. The medium was changed every 2 days, and the cells were split when they reached 80–90% confluence.

MSCs were phenotypically characterized by flow cytometry (FACSVerse, BD Biosciences) using the BD Stemflow™ hMSC Analysis Kit and HLA-DR-PE antibody, following the manufacturer’s instructions (Pharmingen, BD Biosciences, Franklin Lakes, NJ, USA). Ten thousand events were recorded for each sample, and data was analyzed using the FlowJo software 10.0.7 (Treestar Inc.). MSCs from the 4th to 6th passage were used for experiments.

### MSC priming

MSCs were cultured for 24 h in cell culture media lacking FBS supplemented with 15% plasma from each individual GVHD samples or with 15% pooled plasma from healthy donors. Then, the cells were washed with PBS for three times before the beginning of the experiments.

### MSC proliferation

The effect of GVHD plasma over MSCs proliferation was accessed directly by cell count in hemocytometer. Briefly, 2X103 MSCs were plated in 96 well plates and primed with GVHD plasma or pooled plasma obtained from healthy donors (control). At the 3rd and 5th days post priming, cells were detached by trypsinization and counted using a hemocytometer to determine the number of MSCs.

### Immunosuppression assay

For the immunosuppression assay, peripheral blood mononuclear cells (PBMCs) were obtained from healthy volunteers. Such cells were activated with 10 μg/mL of phytohaemagglutinin (PHA, Sigma-Aldrich, St. Louis, MO, USA) and stained with 2.5 μM carboxyfluorescein succinimidyl ester (CFSE), as previously described [25]. After isolation, PBMCs were co-cultured for 5 days with MSCs primed with healthy plasmas (control) or with MSCs primed with GVHD plasmas (10:1 ratio). Then, PBMCs were recovered and stained with anti-CD3, and the proliferation of T cells was determined by flow cytometry.

### MSC morphology

In order to assess whether GVHD plasma priming would alter MSC morphology, MSCs primed with healthy plasmas (control) and MSCs primed with GVHD plasmas were analyzed according to their morphology following the priming protocol described before. Cells from 6 different areas of the well were photographed. Feret diameter, cytoplasm, and nucleus area, as well as cell perimeter, were quantified using the ImageJ software.

### Generation of regulatory T cells

MSCs promote the generation and expansion of regulatory T cells (Tregs) to control the immune response [23]. In order to determine if the MSC priming with GVHD plasma has any influence on this process, primed cells were cultured with phytohemagglutinin (PHA)-activated PBMCs for 5 days. Then, PBMCs were recovered and stained with anti-CD4-FITC, anti-CD25-APC, and FOXP3-PE, according to the manufacturer’s recommendations (FoxP3 Staining Kit, BD Pharmingen). Fifty thousand events were recorded for each sample, and data were analyzed using FlowJo software 10.0.7.

### VCAM-1 and ICAM-1 expression

Considering that ICAM-1 (CD54) and VCAM-1 (CD106) have a crucial role in the immunoregulatory mechanisms of MSCs [2, 3], we investigate the expression profile of these proteins in primed MSCs. MSCs primed with healthy plasmas (control) and MSCs primed with GVHD plasmas were stained with anti-CD54 (conjugated with allophycocyanin (APC)), anti-CD106 (conjugated with fluorescein isothiocyanate (FITC)), or isotype controls (eBioscience, San Diego, CA, USA). After incubation, CD54 and CD106 expressions were determined by flow cytometry. Ten thousand events were recorded for each sample, and data were analyzed using FlowJo software 10.0.7.

### Real-time PCR

TGF-β (Hs00998133), IDO (Hs00984148), PDL-1, IL-10 (Hs00961622), COX-2, HGF, and Galectin-1 (Hs00355202) mRNA expression were analyzed in MSCs primed with healthy plasmas (control) and MSCs primed with GVHD plasmas. Basically, after the priming, MSCs were recovered, and their RNA was obtained using the PureLink RNA Mini Kit, as indicated by the manufacturer (ThermoFisher, USA). The RNA amount and quality were determined using NanoDrop 1000 spectrophotometer (NanoDrop, Wilmington, DE, USA). Total RNA was reverse transcribed using the High Capacity cDNA Reverse Transcription Kit, and real-time PCR was performed using either TaqMan probes and GoTaq qPCR Master Mix (Promega, USA) or SYBR Green Master Mix (Thermo Fisher, USA) combined with primers specific to each gene. Amplification reactions were performed in duplicates, and the relative fold value was obtained by the 2−ΔΔCt method. Data were normalized according to the GAPDH mRNA expression, as well as to the average Ct of the control MSC group. The primer sequences were as follows: PDL-1 sense primer sequence, 5′-AAACAATTAGACCTGGCTG-3′; PDL-1 antisense primer sequence, 5′-TCTTACCACTCAGGACTTG-3′; COX-2 sense primer sequence, 5′-GAAGTTGGCAGCAAATTGAGC-3′; COX-2 antisense primer sequence, 5′-TTCTCCTGTGAAGGCGATGA-3′; HGF sense primer sequence, 5′-CATGCTGGCCCTTACCTAGC-3′; and HGF antisense primer sequence, 5′-GAGGAGAGGACCAAGTTCACA-3′.

### Statistical analysis

Statistical analyses were performed using the Prism 7 software (GraphPad Software Inc., San Diego, CA, USA). The statistical significance was calculated using *t* test analyses to compare the differences between the two groups and using ANOVA to compare three or more experimental groups. Spearman analysis was performed to explore the correlation among the results of the measured variables. The results are presented as the mean ± SD of three independent experiments. The value of *p* < 0.05 was considered statistically significant.

## Results

### Clinical characteristics of GVHD patients

Nineteen patients (12 males and 7 females) between 10 and 70 years of age (mea*n* = 35 years) were included in this study. Their diagnoses included aplastic anemia (*n* = 3), acute lymphoblastic leukemia (*n* = 5), biphenotypic acute leukemia (*n* = 2), chronic myeloid (*n* = 4), acute myeloid leukemia (*n* = 4), and myelofibrosis (*n* = 1). After bone marrow transplantation, 7 patients developed aGVHD, and 12 developed cGVHD. More detailed characteristics of the patients are listed in Table 1.

**Table 1:** Clinical characteristics of patients with GvHD

GvHD classification	Sample	Age	Sex	Diagnosis	SCT source	GvHD affected organ/score	GvHD prophylaxis
Acute	GA1	14	M	AA	BM	Skin I	SIRO + MMF
	GA2	10	F	ALL	BM	Skin III	SIRO + MMF
	GA3	44	F	AA	BM	Eyes I/mouth I	–
	GA4	30	F	B-ALL	BM	Mouth I/liver I	SIRO
	GA5	19	M	ALL	BM	Skin/liver^b^	Prednisone
	GA6	21	M	AA/PNH+	BM	Skin IV/liver/GI^b^	Prednisone + MMF
	GA7	12	M	B-ALL	BM	Skin III	CSA
Chronic	GC1	26	M	BAL/MRD+	BM	Mild	–
	GC2	36	M	CML	BM	Mild	–
	GC3	52	F	AML	PBSC	–	CSA + prednisone
	GC4	41	F	BAL/MRD+	PBSC	Moderate	CSA
	GC5	50	F	CML	BM	–	CSA
	GC6	43	M	CML	PBSC	Moderate	CSA + prednisone
	GC7	12	M	ALL	BM	Mild	–
	GC8	24	F	AML	PBSC	Mild	–
	GC9	70	M	AML	BM	Mild	CSA
	GC10	69	M	AML	BM	Severe	CSA
	GC11^a^	61	M	Myelofibrosis	PBSC	Moderate	CSA + MMF
	GC12	43	M	CML	PBSC	Severe	–

*M* male, *F* female, *AA* aplastic anemia, *ALL* acute lymphoblastic leukemia, *AML* acute myeloblastic leukemia, *BAL* biphenotypic acute leukemia, *BM* bone marrow, *CML* chronic myeloid leukemia, *CSA* cyclosporine, *MMF* mycophenolate mofetil, *MRD* minimal residual disease, *PBSC* peripheral blood stem cells, *PNH* paroxymal nocturnal hemoglobinuria, *SCT* stem cell transplantation, *SIRO* sirolimus

^a^Overlap syndrome

^b^Non-classified

### Inflammatory profile of GVHD plasmas

**Fig. 1:**
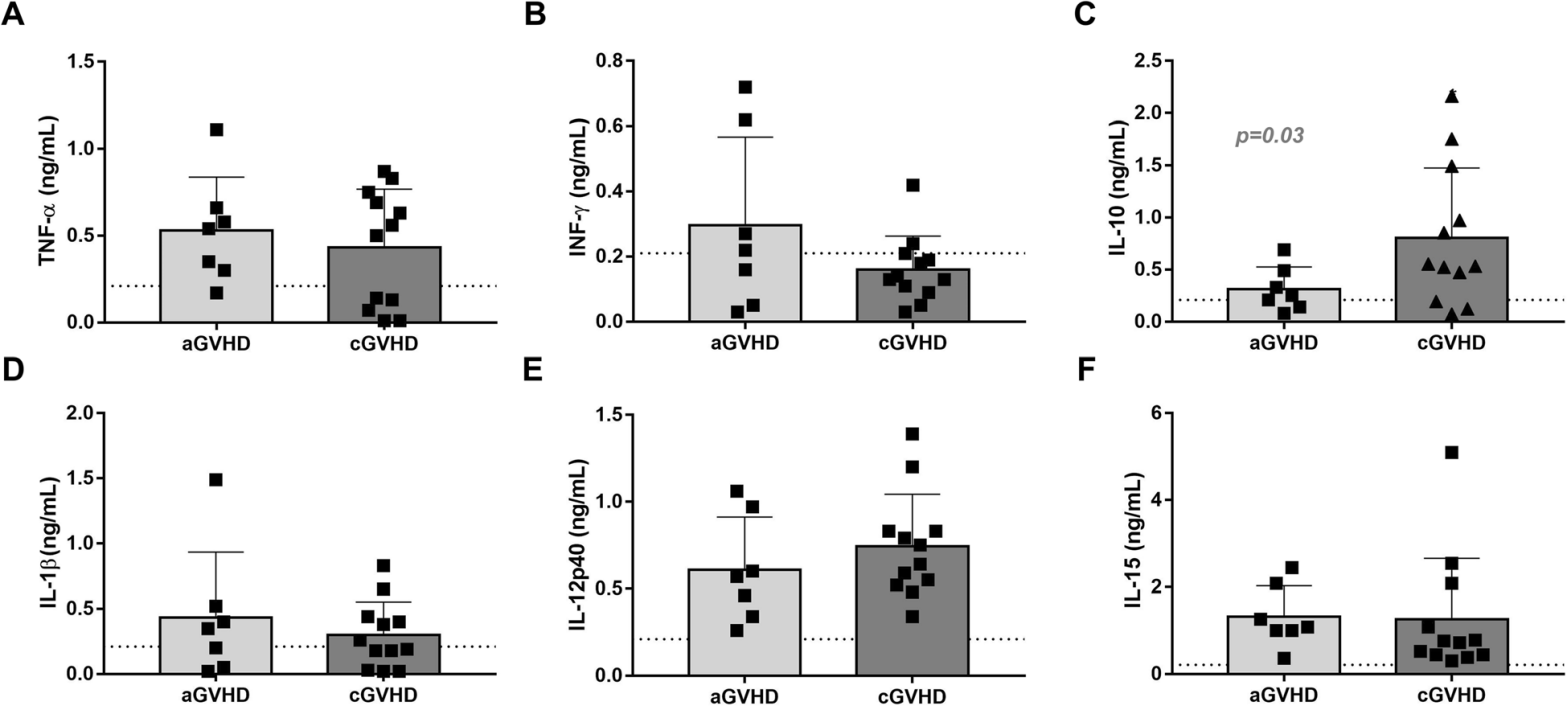
Quantification of immunomodulatory cytokines in aGVHD and cGVHD plasmas. **a**–**f** The levels of TNF-α, IFN-γ, IL-10, IL-1β, IL-12p40, and IL-15 were assessed by ELISA in 7 samples of aGVHD, 12 samples of cGVHD, and also in a plasma pool comprising samples obtained from 8 healthy donors (represented by the horizontal line). Results are presented as mean concentration ± SD. aGVHD, acute GVHD; cGVHD, chronic GVHD

The levels of TNF-α, IFN-γ, IL-10, IL-1β, IL-12p40, and IL-15 were determined in the aGVHD and cGVHD plasma samples. The level of such soluble factors was markedly heterogeneous between the patient samples, and we did not detect statistically significant differences between the cGVHD and aGVHD plasmas, except for IL-10, which was present in higher concentration in the cGVHD samples (*p* = 0.03) (Fig. 1).

### MSC phenotype was not altered by GVHD plasma priming

**Fig. 2:**
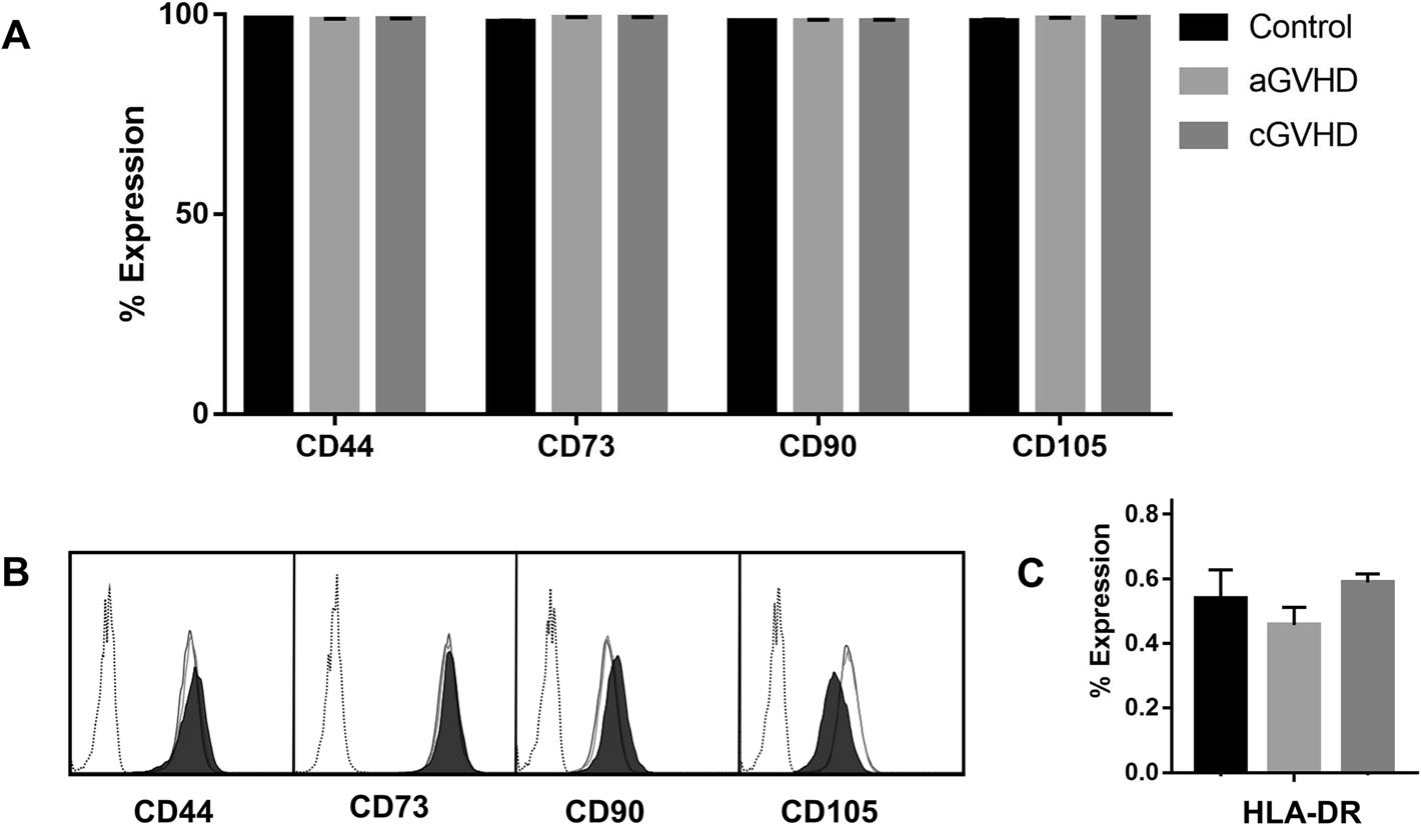
Immunophenotypic characterization of MSCs. **a** The expression of CD44, CD73, CD90, and CD105 was analyzed in control (MSCs primed with pooled plasma from healthy donors), as well in MSCs primed with aGVHD (acute GVHD) and cGVHD (chronic GVHD) by flow cytometry. **b** Representative histograms of single MSC samples investigated in each experimental group. **c** HLA-DR expression in control MSCs, as well as in MSCs primed with aGVHD (acute GVHD) and cGVHD (chronic GVHD)

MSCs primed with GVHD plasmas and MSCs primed with healthy plasmas (control) presented similar immunophenotype, with positive expression of CD44, CD73, CD90, and CD105 (Fig. 2a, b). And no difference was detected regarding HLA-DR expression in those cells (Fig. 2c).

### MSC proliferation after priming with GVHD plasma

**Fig. 3:**
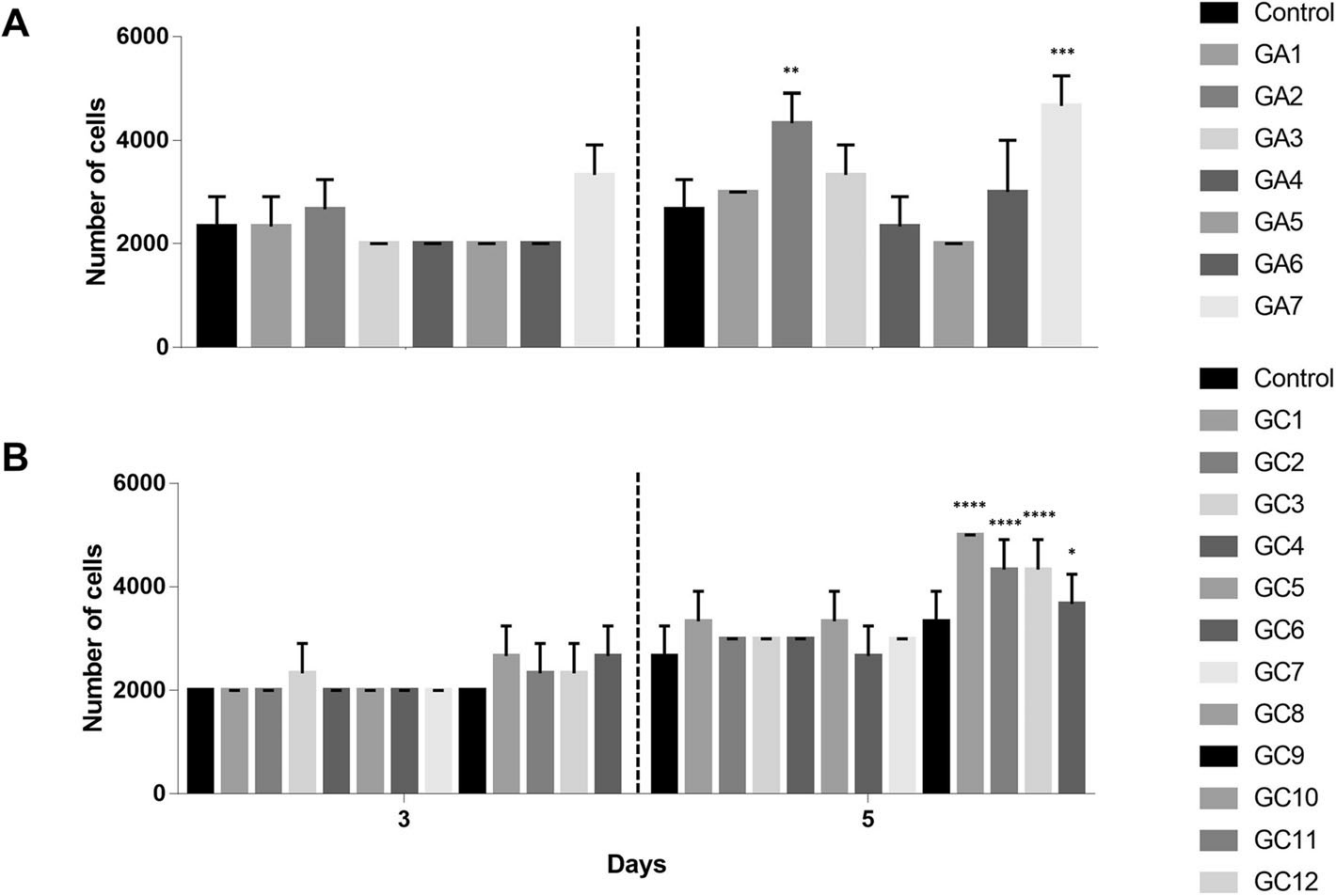
MSC proliferation. **a** Number of MSCs cultured in the presence of either a pool of plasmas obtained from healthy donors (control) or individual aGVHD plasma samples GA1-GA7. **b** Number of MSCs cultured in the presence of control and individual cGVHD (GC1-GC12) plasma samples. The number of MSCs was determined after 3 and 5 days of cell culture. Results are presented as mean ± SD of three independent experiments. **p <0.01 and ***p <0.001

Priming of MSCs with aGVHD or cGVHD did not change the proliferative capacity of MSCs after 3 days of culture. On the 5th day of culture a higher proliferative capacity was observed in MSCs primed with GA2 (p = 0.006), GA7 (p = 0.0006), GC9 (p =0.0001), GC10 (p = 0.0001), GC11 (p = 0.0001), and GC12 (p=0.03), compared to MSCs which were primed with plasmas obtained from healthy donors (control) (Fig. 3).

### MSC priming with specific plasma samples boosted their immunosuppressive capacity

**Fig. 4:**
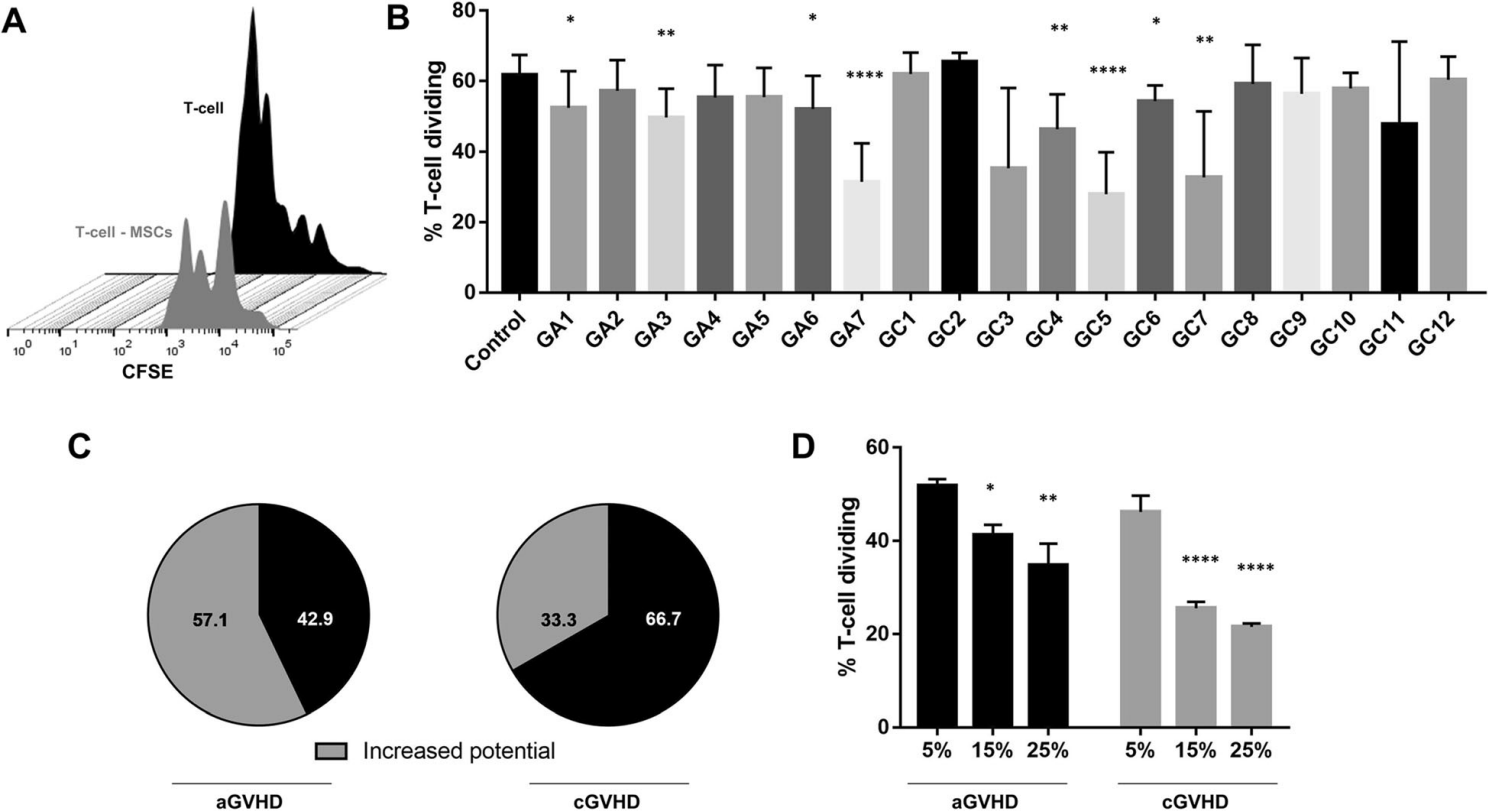
Immunosuppressive potential of MSCs. **a** Representative CFSE histogram of activated T cell (black histogram) and activated T cell co-cultured with MSCs (T cell—MSCs, gray histogram). **b** T cell proliferation after co-culture with control MSCs (primed with pooled plasma from healthy donors) and MSCs primed with acute GVHD (GA 1-7) and chronic GVHD (GC 1-12) individual plasma samples. **c** Percentage of aGVHD and cGVHD plasmas which succeeded or failed to increase the immunosuppressive potential of primed MSCs. **d** T cell proliferation after co-culture with 5%, 15%, and 25% of the pool of aGVHD and cGVHD plasmas that enhanced the MSC immunosuppressive function. Results are presented as mean concentration ± SD of three independent experiments. *p < 0.05, **p < 0.01, and ****p < 0.0001

As expected, MSCs used in this study were able to inhibit T cell proliferation (*p* < 0.0001) (Fig. 4a). More importantly, 57% of the aGVHD and 33% of the cGVHD plasmas tested to prime MSCs were able to potentialize the immunosuppressive effect of these cells. The plasma samples which promoted superior immunosuppressive capacity of MSCs were GA1 (*p* = 0.04), GA3 (*p* = 0.006), GA6 (*p* = 0.02), GA7 (*p* < 0.0001), GC3 (*p* = 0.009), GC4 (*p* = 0.003), GC5 (*p* < 0.0001), GC6 (*p* = 0.01), and GC7 (*p* = 0.002) (Fig. 4b, c).

Considering these findings, we prepared two separate pools, including the aGVHD and cGVHD plasmas, which were able to enhance the immunosuppressive potential of MSCs. MSCs were primed with such plasma pools, which were tested at 5, 15, and 25%. Importantly, we noticed a dose-response pattern, in which an increased immunosuppressive potential in MSCs primed with aGVHD pool at 15% (*p* = 0.01) and 25% (*p* = 0.001) was observed when compared to cells primed with aGVHD at 5%. The same pattern was observed for the MSCs primed with cGVHD pool. Compared to cells primed with 5% cGVHD pool, the cells primed with 15% (*p* < 0.0001) and 25% (*p* < 0.0001) plasma pools presented a higher capacity to control T cell proliferation (Fig. 4d).

Due to their pronounced efficacy in boosting MSC immunomodulatory capacity, GA1, GA3, GA6, GA7, GC3, GC4, GC5, GC6, and GC7 were selected for further experiments.

### Primed MSCs with enhanced immunosuppressive potential show altered morphology

**Fig. 5:**
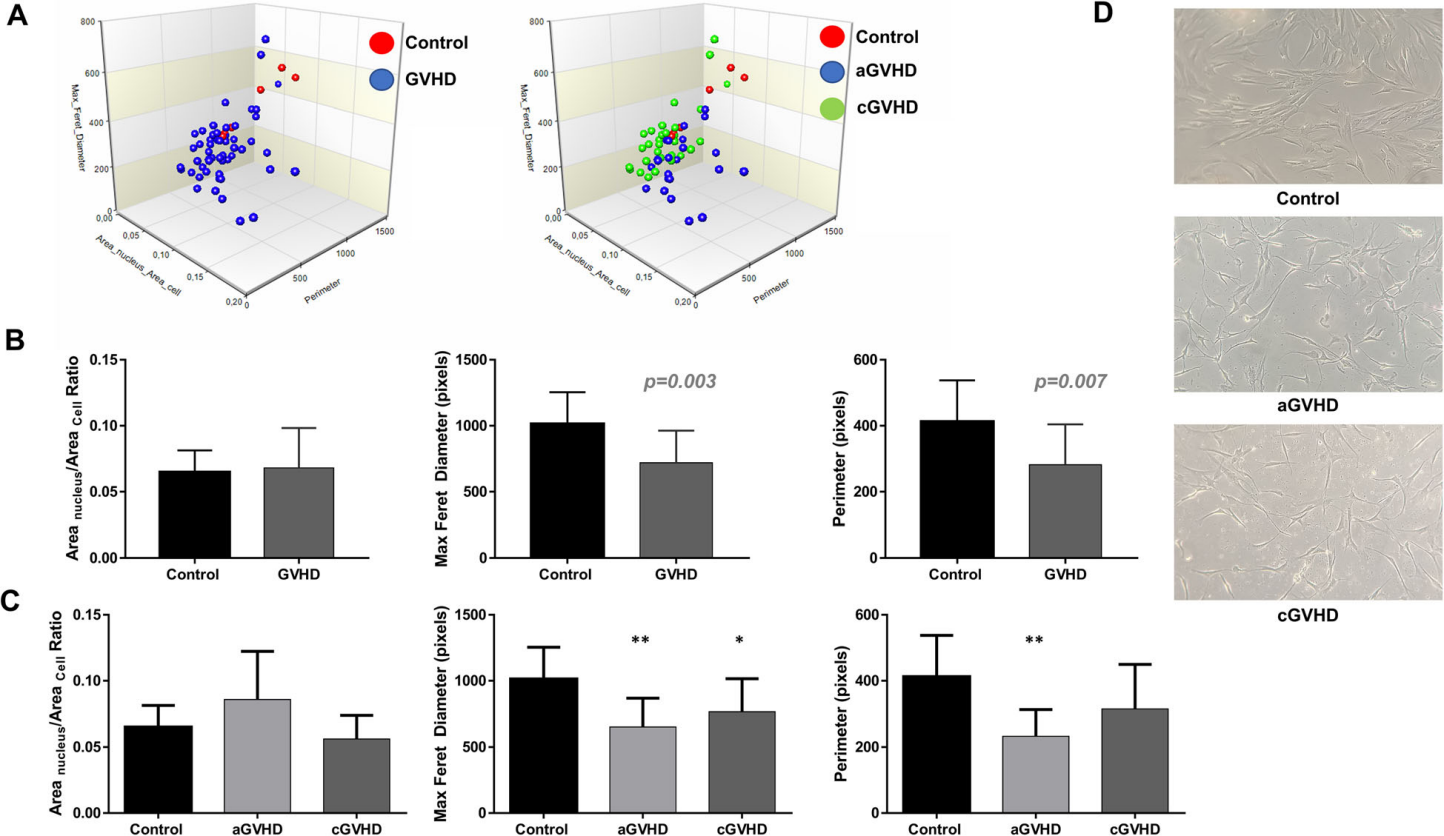
Morphometric evaluation of MSCs. MSCs primed with pooled plasma from healthy donors (control) and primed with GVHD plasmas were analyzed according to the nucleus and cytoplasmic area ratio, maximum Feret diameter, and perimeter. **a** 3D graph showing the different distribution of control and MSCs primed with GVHD plasmas. **b** Nucleus and cytoplasmic area ratio, maximum Feret diameter, and perimeter comparisons between the control and MSCs primed with GVHD plasmas. **c** The nucleus and cytoplasmic area ratio, maximum Feret diameter, and perimeter analysis of control MSCs, as well as MSCs primed with aGVHD and cGVHD plasmas. **d** Representative morphology of MSC primed with pooled plasma from healthy donors (control), aGVHD, and cGVHD plasmas (magnification × 100). Results are presented as means or means ± SD of three independent experiments. *p < 0.05 and **p < 0.01

It has been described that MSCs with the highest immunosuppressive potential have specific morphological features, i.e., a higher ratio of nucleic/cytoplasmic area, smaller perimeter, and Feret diameter. Those characteristics can be used as a parameter to assess the MSC immunosuppressive potential [28, 29]. In order to investigate whether GVHD plasma priming would alter MSC morphology, MSCs primed with GVHD plasmas and MSCs primed with healthy plasmas (control) were analyzed immediately after the priming protocol. Corroborating the data obtained from the immunosuppression assay, the MSCs primed with GVHD plasmas presented a morphological pattern indicative of higher immunosuppressive potential. Despite presenting a similar ratio of nucleic/cytoplasmic area, the MSCs primed with GVHD plasmas showed smaller maximum Feret diameter (*p* < 0.01) and perimeter (*p* < 0.05) (Fig. 5a, b). When MSCs primed with aGVHD and cGVHD samples were analyzed separately, it was possible to observe that aGVHD samples had even smaller maximum Feret diameter and perimeter compared to control and cGVHD primed MSCs (Fig. 5c).

### Generations of Tregs by MSCs

**Fig. 6:**
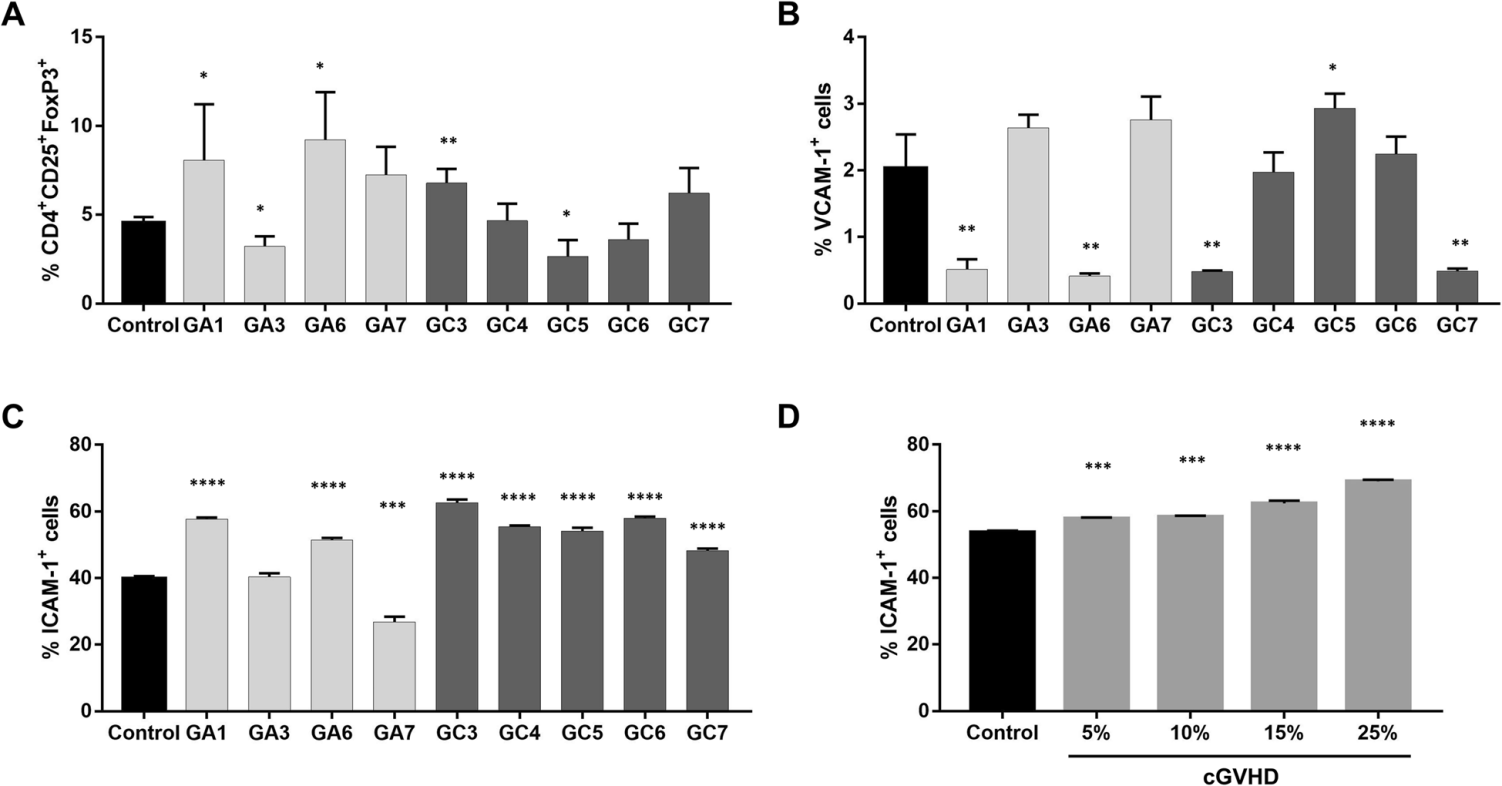
Generation of Tregs and expression of adhesion molecules on MSC surface. **a** The percentage of CD4^+^CD25^+^FOXP3^+^ in PBMCs cultured with control MSCs and MSCs primed with aGVHD plasmas (GA1, GA3, GA6, GA7) and cGVHD (GC3, GC4, GC5, GC6, GC7) was assessed by flow cytometry. This technique also was used to determine the expression of VCAM-1 (**b**) and ICAM-1 (**c**) on control and primed MSCs. **d** ICAM-1 expression was determined also in MSCs primed with crescent concentrations of a pool of cGVHD plasmas. Results are presented as mean concentration ± SD of three independent experiments. *p < 0.05, **p < 0.01, ***p < 0.001, and ****p < 0.0001

One of the several mechanisms of MSC immunosuppression is the induction of differentiation and expansion of Tregs. Here, we noticed that MSCs primed with GA1 (*p* = 0.05), GA6 (*p* = 0.01), and GC3 (*p* = 0.005) promoted Treg generation, while those primed with GA3 (*p* = 0.01) and GC5 (*p* = 0.01) actually impaired the production of such cells (Fig. 6a).

### MSCs primed with cGVHD present increased expression of ICAM-1

We then interrogated whether priming MSCs with GVHD plasmas exerted any impact over the expression of VCAM-1 and ICAM-1. We observed that only MSCs primed with GC5 plasma presented increased expression of VCAM-1 (*p* = 0.02), while MSCs primed with GA1 (*p* = 0.003), GA6 (*p* = 0.002), GC3 (*p* = 0.003), and GC7 (*p* = 0.003) plasmas presented a decreased expression of such adhesion molecule (Fig. 6b). Regarding ICAM-1 expression, while GA7 (*p* = 0.0001) decreased the expression of this molecule on primed MSCs, GA1 (*p* < 0.0001) and GA6 (*p* < 0.0001) increased this expression. Surprisingly, all cGVHD plasmas tested promoted ICAM-1 hyperexpression on MSC surface (*p* < 0.0001) (Fig. 6c). In order to validate this finding, we promoted MSCs priming with increasing concentrations of pooled cGVHD plasmas (5, 10, 15, and 25%), and it was noticed that such pool induced the hyperexpression of ICAM-1 in a dose-dependent manner (Fig. 6d).

### Gene expression of MSCs primed with GVHD plasma

**Fig. 7:**
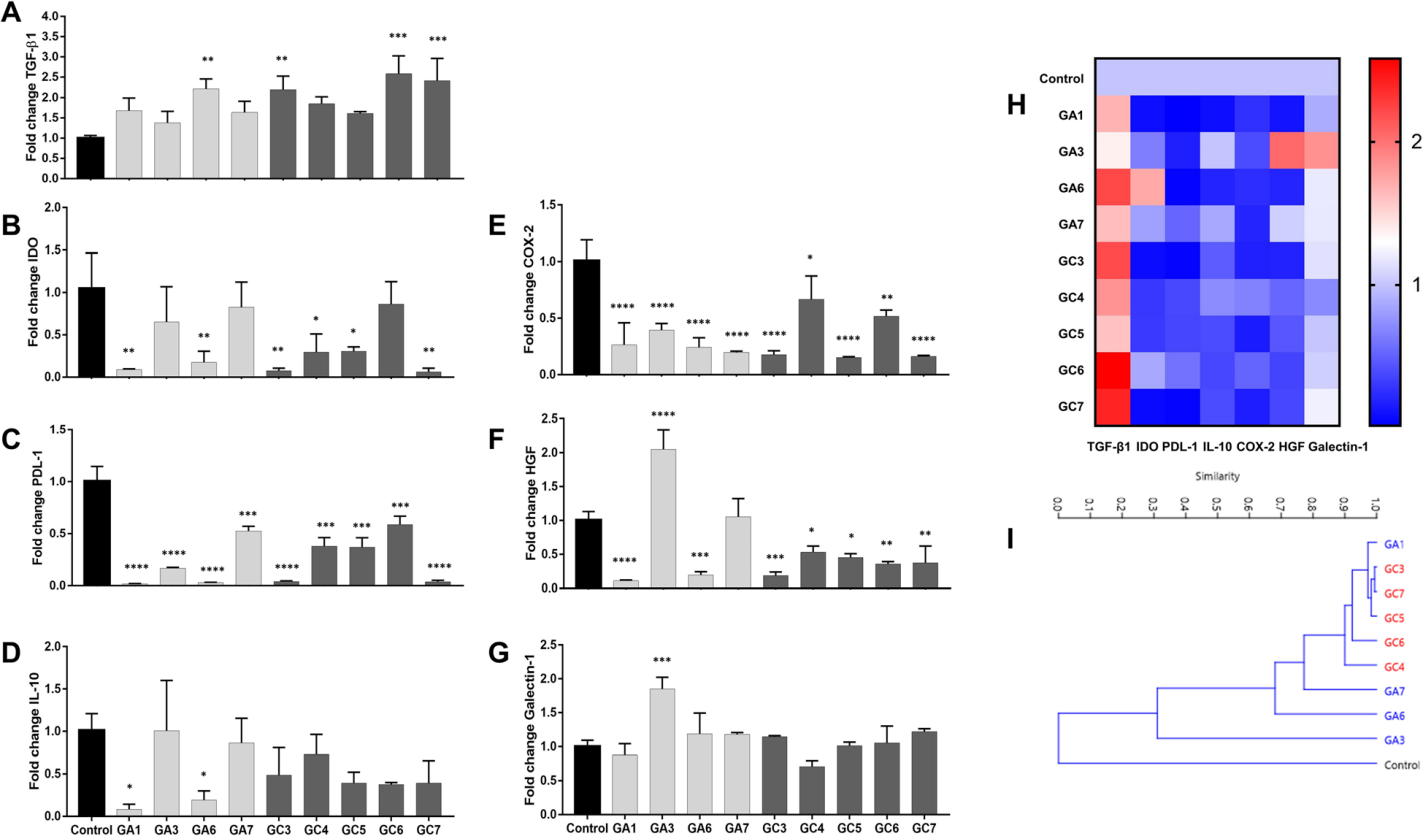
Gene expression analysis of selected transcripts. **a**–**g** TGF-β, IDO, PDL-1, IL-10, COX-2, HGF, and Galectin-1 expression were assessed in MSCs primed with healthy plasmas (control) and in MSCs primed with the plasmas GA1, GA3, GA6, GA7, GC3, GC4, GC5, GC6, and GC7. **h** Heat map of specific genes expressed in control MSCs and primed MSCs. **i** Hierarchical clustering of gene expression data. *p < 0.05, **p < 0.01, *** *p* < 0.001, and ****p < 0.0001

mRNA expression of TGF-β, IDO, PDL-1, IL-10, COX-2, HGF, and Galectin-1 were assessed in MSCs primed with healthy plasmas (control) and in MSCs primed with GVHD plasmas. TGF-β transcriptional levels were increased in MSCs primed with GA6 (*p* = 0.003), GC3 (*p* = 0.003), GC6 (*p* = 0.0001), and GC7 (*p* = 0.0006). It is important to note that although no statistical difference was detected, all other GVHD plasmas tested as a priming strategy also increased the TGF-β expression by at least 37%. None of the tested plasmas were able to induce increased expression of IDO and IL-10 in primed MSCs, compared to the control counterparts. Also, all GVHD plasmas tested caused a reduction in PDL-1 and COX-2 expression by MSCs. Only plasma GA3 promoted greater HGF (*p* < 0.0001) and Galectin-1 (*p* < 0.0001) expression compared to control samples (Fig. 7a–g). Despite individual variability among samples, hierarchical clustering of the transcriptional profile of primed MSCs revealed a higher similarity among MSCs primed with cGVHD, compared to those primed with aGVHD.

### Correlation analysis

**Fig. 8:**
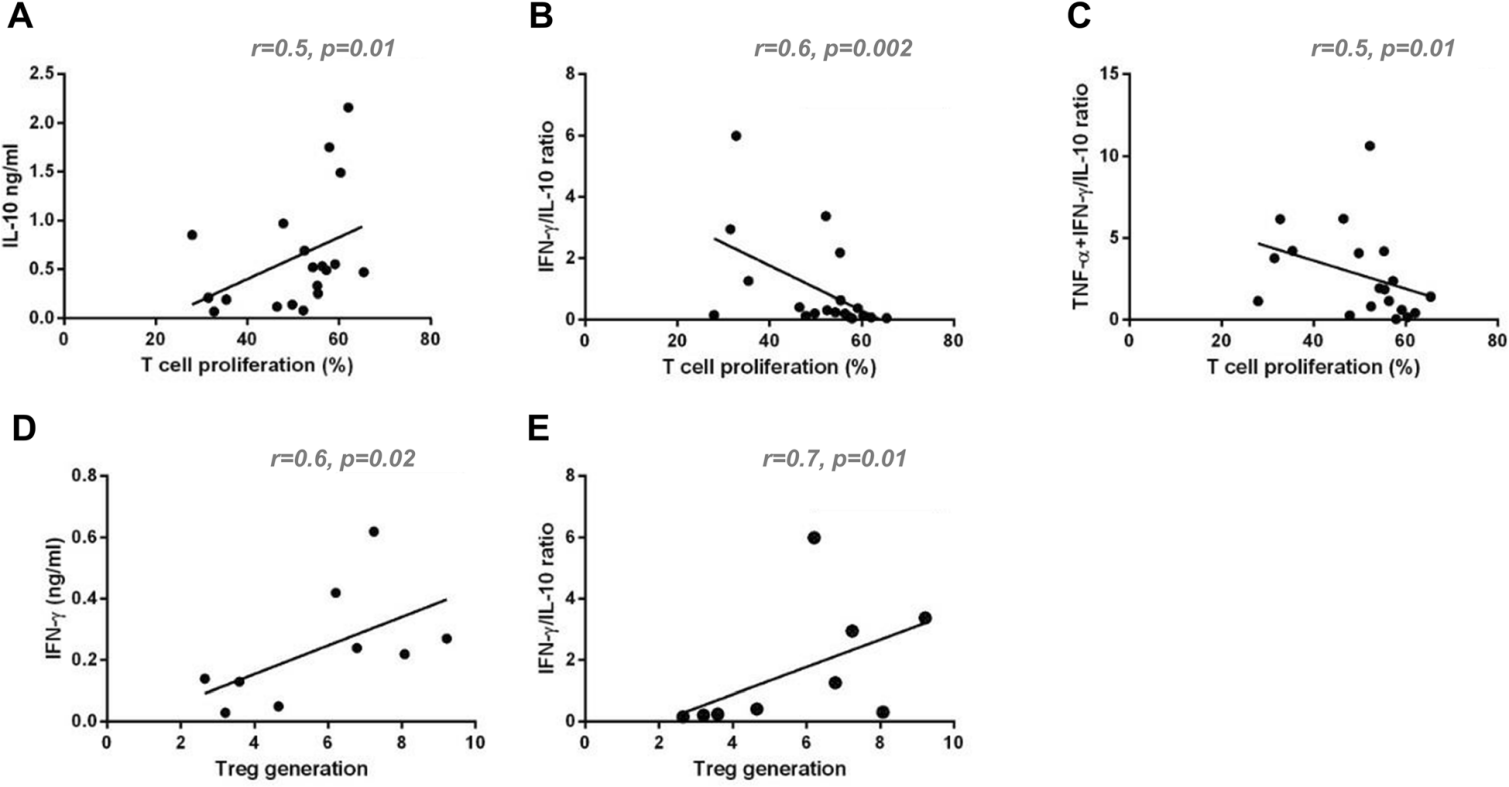
Correlation analysis between plasma cytokine levels and primed MSC immunosuppressive behavior. The levels of IL-10 (**a**), IFN-γ (**d**), the ratio of IFN-γ/IL-10 (**b**, **e**), and the ratio of (TNF-α+IFN-γ)/IL-10 (**c**) cytokines in the plasmas used to prime MSCs were significantly associated with the capacity of primed MSCs to control T cell proliferation (**a**–**c**) and to promote Treg generation (**d**, **e**), regardless of the GVHD disease type

Since the parameters analyzed presented high variability between patients, we decided to investigate how plasma components used for MSC priming might influence the MSC-related variables. First, we analyzed how different parameters correlated to the percentage of T cell proliferation (Fig. 8a–c). Interestingly, we observed that relatively high IL-10 levels in the plasma used for MSC priming were associated with a lower immunosuppressive capacity of primed MSCs (*r* = 0.50, *p* = 0.01), regardless of the IFN-γ levels in the tested plasmas. Considered as a discrete variable, IFN-γ was not significantly correlated with primed MSC capacity to inhibit T cell proliferation (*r* = 0.30, *p* = 0.10). However, when the ratio between IFN-γ/IL-10 plasma levels was considered, a significant correlation was observed; the higher the IFN-γ/IL-10 ratio, the more effective the primed cells were to inhibit T cell proliferation (*r* = − 0.60, *p* = 0.02). A similar observation was made when the sum of IFN-γ and TNF-α content divided by IL-10 levels in the plasma were analyzed (*r* = − 0.52, *p* = 0.01). Finally, we noticed that both high IFN-γ and also high IFN-γ/IL-10 ratio in the plasma led to a higher capacity of primed MSCs to induce Treg generation (*r* = 0.66, *p* = 0.02, and *r* = 0.71, *p* = 0.01, respectfully) (Fig. 8a–e).

## Discussion

Recently, several studies have been published demonstrating that the immunosuppressive functions of MSCs can be boosted by priming with recombinant inflammatory factors, especially IFN-γ, TNF-α, and IL-17 [22, 23]. With this in mind, in this study, we interrogated whether the use of the plasma collected from GVHD patients might be an effective and innovative strategy to boost MSC immunomodulatory behavior. Importantly, our data have shown that GVHD-derived plasma can be used as a biological alternative to enhance the immunosuppressive potential of MSCs.

A storm of inflammatory factors has been described to occur during GVHD, arising primarily from the radiation and chemotherapy conditioning damage, but also influenced by patient microbiota and activated immune cells [30]. In order to characterize our GVHD cohort and the biological products used to prime MSCs, the levels of IFN-γ, TNF-α, IL-1β, IL-12p40, IL-15, and IL-10 were determined. Higher levels of IL-10 were observed in cGVHD samples, which constitute a biological marker of this disease [31, 32]. The concentration of the other factors was markedly heterogeneous among the GVHD patients, which may be explained, at least in part, by the different therapeutic regimens of each patient [33].

Interestingly, despite IFN-γ being considered the cytokine with the highest capacity to potentialize MSC immunomodulatory behavior, its levels were not significantly correlated with an enhanced capacity of primed MSCs to inhibit T cell proliferation. Still, IFN-γ levels in the plasma were significantly correlated with a higher capacity of primed MSCs to induce Treg generation. IL-10 levels in the plasmas used to prime MSCs were significantly correlated with a higher capacity of primed MSCs to control T cell proliferation. The ratio of IFN-γ/IL-10 cytokines in the plasma presented the highest significance in the correlation with T cell proliferation inhibition by primed MSCs and also Treg generation. Such observations allow to hypothesize that, in the presence of a more suppressive milieu (i.e., incubation with high levels of IL-10), MSCs are not adequately primed licensed to control the immune response, while in a more inflammatory milieu, defined not only by IFN-γ but also by IL-10 cytokine levels, the primed MSCs potentialize their capacity to inhibit T cell proliferation and to promote Treg generation. Such data also allows hypothesizing that IFN-γ and IL-10 cytokine levels differ between GVHD patients who respond or not to MSC therapy, which could help explain, at least in part, the observed outcomes. We propose that such hypotheses should be tested, in order to confirm whether it would be possible to boost MSC immunomodulation by priming MSCs in high IFN-γ/IL-10 environments and also to screen patients (and therapeutic windows) who would most probably benefit from MSC therapy.

In the present study, the primed MSCs were also investigated in order to understand how the priming strategies altered their phenotype. First, both aGVHD and cGVHD MSC priming were phenotyped and shown to maintain the classical profile of MSCs. Importantly, none of these priming strategies investigated induced the expression of HLA-DR in MSCs, which may represent an advantage in relation to priming with IFN-γ [34]. Interestingly, the GVHD plasmas were also shown to enhance the MSC proliferative capacity. More importantly, we identified that some aGVHD and cGVHD plasmas were able to strongly enhance the immunomodulatory capacity of MSCs, possibly in a dose-dependent manner, as shown in the pooled plasma experiments.

Even though Leijs and colleagues were not investigating MSC priming strategies, they reported that synovial fluid from arthritis patients enhances IDO expression and the immunoregulatory capacity of MSCs [35]. In contrast to their findings, our data revealed that primed MSCs displaying increased immunosuppressive function generally presented variable IDO, IL-10, and Galectin-1 mRNA levels when compared to control samples. Nevertheless, we observed an increased expression of TGF-β transcripts in primed MSCs, which is an important mediator of the immunosuppressive role of MSC over both innate and adaptive immunity cells [4, 36]. Surprisingly, even though TGF-β participates in the generation of Tregs by MSCs [37], we did not notice a greater production of Tregs by primed MSCs.

The adhesion molecule ICAM-1 is an important mediator of the immunosuppressive function of MSCs. It was demonstrated that the expression of this adhesion molecule could be enhanced by the presence of IFN-γ and TNF-α [38]. Surprisingly, all cGVHD samples that enhanced the immunosuppressive function of MSCs induced a hyperexpression of ICAM-1 on their surface, even though these plasmas did not show the highest levels of ICAM-1. Interestingly, though, this effect was gradually increased as MSCs were primed with higher concentrations of cGVHD plasma. Recently, Tang and colleagues demonstrated that MSCs overexpressing ICAM-1 prolonged the survival of mice with GVHD [39]. In accordance with such observation, MSCs overexpressing ICAM-1 were shown to possess stronger therapeutic effects than ICAM-1-low MSCs in a mouse model of inflammatory bowel disease [40].

Besides the altered gene and protein expression in primed MSCs, we also observed a marked alteration in cellular morphology. Therefore, we decided to quantify such data and realized that, as shown by Klinker and colleagues [28], as well as Marklein and colleagues [29], MSCs with the highest immunomodulatory capacity are relatively smaller than their counterparts, presenting a higher nucleus to the cytoplasmic area, lower perimeter, and maximum Feret diameter.

A significant limitation of our study is the fact that the plasmas were obtained from GVHD patients that presented different bone marrow diseases and different GVHD scores and received different therapeutic regimens. Since previous reports indicate that immunosuppressants can affect MSC functions [41, 42], we cannot rule out the possible contribution of these drugs to the effects observed in our study.

## Conclusions

To the best of our knowledge, the present work is the first to demonstrate the use of a biological product as a strategy to prime MSCs, in which the patient’s own disease is explored. Overall, we hope our observations will pave the way for further validation in preclinical and clinical models. The obtained results may aid the development of new therapeutic approaches for the treatment of GVHD and other immune diseases.

## Data Availability

The authors confirm that all data underlying the findings are fully available.

## Abbreviations

GAL-1: Galectin 1
GAL-9: Galectin 9
GVHD: Graft-versus-host disease
HGF: Hepatocyte growth factor
ICAM-1: Intercellular adhesion molecule 1
IDO: Indoleamine 2,3-dioxygenase
IL-1β: Interleukin-1 beta
IL-6: Interleukin-6
IL-10: Interleukin-10
IL-12p40: Interleukin-12
IL-15: Interleukin-15
IFN-γ: Interferon gamma
MSC: Mesenchymal stem cell
PBMC: Peripheral blood mononuclear cells
PDL-1: Programmed death-ligand 1
PGE2: Prostaglandin E2
TGF-β: Transforming growth factor beta
TNF-α: Tumor necrosis factor alpha
Treg: Regulatory T cell
VCAM-1: Vascular cell adhesion protein 1
